# Tetanus in War: Value of the Serum Treatment

**Published:** 1915-10-16

**Authors:** 


### TETANUS IN WAR.

### VALUE OF THE SERUM TREATMENT.

By causing a large increase in the opportunities for tetanus infection, the war has brought into greater prominence than they might otherwise have attained the researches of the last two or three years on the serum treatment of that disease. These researches, and their practical upshot, are object-lessons in more than one way. They provide new facts and materials so damaging to the position of anti-vivisectionists and similar fanatics that the propaganda for the prevention of medical progress is less likely than ever to attract any general public support. They show, also, that initial failure, or comparative failure, with methods of serum therapy in any given disease should not deter scientists from further investigations or from the hope of ultimate success. And they also show that even in the case of diseases whose bacterial causation has been long recognised and established patient research will often show that some factors in the etiology have been overlooked or misunderstood, and that the fullest knowledge of every point bearing on the disease is necessary for the proper direction of research in serum therapy.

Let us see how these principles apply to tetanus, more familiar to many under its realistically terrible name of lock-jaw. It has been known for centuries that this dreadful disease, more often than not fatal in its results, is liable to follow any wound into which dirt, dust, soil, or earth has obtained entrance; though it is only in a minute percentage of such wounds that tetanus develops. The impression is widespread in this country that a cut on the web which separates the thumb from the palm of the hand is especially likely to be followed by lock-jaw. There is no scientific foundation for this belief, though it has been suggested that it may have arisen from an alleged peculiar liability of gardeners to cut themselves in that situation while pruning, and from the frequency with which gardeners' tools are contaminated with earth. Another well-known fact is that in some localities the percentage of mortality is much smaller or greater than the average.

### The Bacillus of Tetanus.

As early as 1884 and 1885 it was thought probable, from the experiments of various scientists, that the disease was specific and infective; that is, due to the activities of a special microbe which could produce tetanus alone, and without which no tetanus could occur. But it was not until 1889 that a Japanese bacteriologist (Kitasato) isolated the bacillus and proved that it was the cause of the disease. According to his researches, the bacillus exists only in the wounded or lacerated parts, thriving there and manufacturing poisonous toxins which are absorbed along the nerves or into the circulation. These toxins are held to have an especially noxious effect upon the brain and spinal cord, and thus to cause the convulsions, cramps, and other symptoms which characterise the disease.

It was early discovered (in 1890 and succeeding years) that an anti-toxin could be obtained from the blood of animals inoculated with non-lethal doses of the tetanus bacillus. High hopes were entertained that the use of this anti-toxin would diminish almost to vanishing point the mortality of tetanus, just as in the case of diphtheria and its anti-toxin, because these two diseases were *par excellence* those in which a local microbe factory was able to poison the system by toxins which spread thence. On this assumption of the likeness between tetanus and diphtheria, cases of tetanus were treated by the injection of anti-toxin under the skin; but the results were disappointing, for no marked improvement in the mortality statistics was perceptible. After that someone propounded the idea that since the tetanus toxin especially attacked the brain, the brain was the part which ought most of all to be bathed in anti-toxin; so about the beginning of this century trephining operations were performed, and the anti-toxin was introduced into the brain through the opening. This, too, failed to give satisfactory results. Later still, the less drastic route of reaching the nervous system by injection into the space round the spinal cord through a spinal cannula was adopted; the results showed but slight improvement, nor did injection into the nerves give any better statistics.

### Preventive Use of the Serum.

As well as the use of tetanus anti-toxin for cases of indubitable, fully developed lock-jaw, scientists also began to urge that it should be given as a *preventive* in cases of wound or injury with obvious fouling of the tissues by earth or mud. For this purpose smaller doses sufficed, and the results were much more definitely beneficial. Thus in the United States there was always a heavy annual death roll from accidents during the celebrations of Independence Day (July 4). In 1903 there were 415 cases of tetanus alone from this source.* The American surgeons took up the question of mitigating the deadly character of Independence Day, and one of their recommendations was the immediate use of tetanus anti-toxin for all patients with fouled wounds on that day. The suggestion was fairly generally acted on, with the result that in 1904 there were but 105 cases of tetanus, and by 1906 the number had fallen to seventy-three. In the following year the editor of the *Journal of the American Medical Association* stated that he could not find in the American literature of tetanus one single case in which that disease had developed in anyone to whom a timely prophylactic dose had been given. Since then many observers have testified to the great value of tetanus anti-toxin as a preventive of tetanus. The doses recommended vary from 500 to 2,000 "U.S.A." units subcutaneously (forty of these units are about equal to one of the German units); an average dose

* MacConkey, *British Medical Journal*, November 10, 1914, from whose paper several of the facts herein cited have been derived.

is 1,000 to 1,500, and higher doses are reserved for severe cases or those in which some days have elapsed before the injection can be given.

Before leaving this topic of prevention by serum, and reverting to treatment of the developed disease, a word may be added about the experience of the British surgeons in the war. During the early weeks of the campaign there were a certain number of cases of tetanus—forty or fifty it was announced, if recollection serves, though popular rumour exaggerated this figure into something a good deal more serious. Then the Army moved from Central France to Flanders, a veritable bogland of the richest and most highly cultivated alluvial soil in Europe. Ordinary expectation was that tetanus would increase by leaps and bounds, especially as the casualty figures became steadily larger. About this time, however, prompt preventive inoculation was introduced and so fully organised that only very rarely did any wounded patient escape it, and the tetanus returns promptly dropped to figures that may be regarded as negligible. An officer who has served in one of the largest base hospitals since the war began is credited with the statement that only one case of tetanus has been seen in that hospital, and that in a man who by some mischance had escaped his preventive dose of serum. No official figures have been published lately, but it is known that tetanus has been almost abolished among the wounded.

Improved Results in Treatment.

So much, then, for the preventive use of antitetanus serum, which is firmly established as one of the most beneficent results of serum therapeutics. Let us turn again to the use of serum in treatment, the results of which were, as has been said above, up to quite lately distinctly disappointing. Within the last four years many researchers have contributed to our knowledge of the tetanus bacillus and the way in which its poisons produce in the body their deadly effects; most of this work has been done on animals, but as they suffer from tetanus in much the same way as human beings, there has been a fair inference that the results apply to us too. It has been shown that to cure an animal, purposely infected with tetanus, it is not enough to give one dose, as is sufficient in diphtheria, and was by analogy supposed to suffice also for tetanus. Repeated doses, and very large doses at that, had been tried by a good many surgeons before the war began, with results decidedly better than were formerly obtained. Nor was one avenue of approach alone selected; serum was given subcutaneously, intramuscularly, intravenously, and intraspinally, all in the same patient. The method advised by MacConkey and taken up by the Army medical authorities consists in the use of an immediate dose of 3,000 to 6,000 units by lumbar puncture and of 5,000 to 20,000 units in the veins as soon as the disease is diagnosed. Then doses of 5,000 to 10,000 units are given subcutaneously or intramuscularly (round the wound itself) every day or every other day until the symptoms entirely abate; and extra doses in the spine or the veins are also given now and then unless the case reacts promptly to the treatment. It will be seen that prompt diagnosis and vigorous treatment are required; and when these are secured there is no doubt that the proportion of recoveries is extraordinarily high—far in advance of anything that has been hitherto obtainable. This treatment does not supplant the need for the old symptomatic treatments, such as absolute quiet, large and frequent doses of sedatives, general anæsthesia for even the most trifling procedures, and stimulants; nor for prompt and drastic surgical treatment of the infected tissues by drainage, excision, irrigation, and so forth. But it does give results that are really worth having and are more than worth the considerable trouble and expense of securing them. On this point the opinion of R.A.M.C. officers with personal experience seems to be unanimous.

